# Epidemiology and Social Inequalities of Periodontal Disease in Brazil

**DOI:** 10.3389/fpubh.2014.00203

**Published:** 2014-10-20

**Authors:** Pamella V. Palma, Isabel Cristina G. Leite

**Affiliations:** ^1^Federal University of Juiz de Fora, Juiz de Fora, Brazil; ^2^Collective Health Department, Federal University of Juiz de Fora, Juiz de Fora, Brazil

**Keywords:** periodontal diseases, epidemiology, health inequalities, Brazil, dental health surveys, oral health

## Introduction

The relationship between social inequality and population health profiles are the subject of studies in oral health, which have shown that both dental caries and periodontal disease are more prevalent in socially disadvantaged individuals. The association with the socio-economic factor, in chronic diseases, reflects risk factors related to the social environment such as smoking and psychosocial stress (1, 2).

The importance of epidemiological studies on oral health, regarding prevalence and severity, rests in the planning of public policies for prevention and care in health services (3). This allows the design of individual and community health strategies for prevention, treatment, and control of periodontal disease, in addition to understanding the levels of demand among people needing periodontal care. In Brazil, there are great many studies related to dental caries in the child population (3, 4). In relation to periodontal health, the number is limited, using various methodologies for case classification, complicating the planning of these actions and comparison of results (2, 5). In international studies, it is noticed that gingivitis and periodontitis are more prevalent in populations with lower socio-economic indicators, such as income and education level (4).

## National Policy on Oral Health – Surveys on Oral Health, 2003 and 2010

Brazil possesses a vast territory, with 8,511,965 km^2^, divided into 26 states and a federal district. These 27 units comprise its five geographic regions, in which ethnic and cultural diversity and socio-economic inequality are quite pronounced. Of the 193,976,530 inhabitants, 8.7% are illiterate and 43% work in an informal way (IBGE, 2012). With a gross domestic product per capita of $11,530, the country exhibits great social inequality. The Gini coefficient for household income was 0.501 in 2011, according to the World Bank (6). The Brazilian health system is public, funded by federal, state, and municipal budgets. It guarantees everyone universal and full access to the actions for health promotion, protection, and recovery. It is estimated that over 80% of the population relies exclusively on the Unified Health System (SUS) for these services (7). However, the decentralized management of the health system may lead to variations in the performance between the various federal units, including oral health services. Inequalities in social parameters and health access were shown in Table 1.

**Table 1:** **Socio-demographic conditions and access to dental services, Brazilian regions, 2014**.

	HDI	Households with basic sanitation	Illiteracy rate	Ratio population/dentist	Number of CEOs	Population without access to dental public services (%)
South	0.771	80.7	5.9	774	57	7.6
Southeast	0.745	83.4	6.6	601	125	8.4
Midwest	0.737	36.0	8.9	747	33	9.7
North	0.664	8.8	11.6	1800	17	17.1
Northeast	0.610	34.5	21.9	1734	104	17.5

In 2004, the Ministry of Health launched the National Oral Health Policy – Brazil Smiling (Brasil Sorridente). Through this measure, oral health care came to be offered in a comprehensive manner, with the intent of using epidemiology and territorial information for planning, and centering actions on the Health Surveillance program, incorporating continuous practices to assess and monitor damage, risks, and determinants of the health-disease process. More complex procedures were included in primary care, and a network of dental care services was created in the SUS (centers of dental specialties – CEO, including periodontal procedures), reclaiming the citizenship of the Brazilian population.

Brazil Smiling had as its epidemiological basis the completion of the SB Brasil 2003 Project – Oral Health Conditions of the Brazilian Population (8), and 7 years after its launch, the Ministry of Health concluded, in 2011, the fourth nationwide epidemiological survey in the Oral Health area, entitled SB Brasil 2010 – National Oral Health Study (9).

The SB Brasil 2003 and 2010 studies were cross-sectional epidemiological studies of oral health on a nationwide basis, with cluster sampling, on different dental problems in children, adolescents, adults, and the elderly in Brazil (8, 9). Their goal was to analyze the Brazilian population’s oral health situation to provide the SUS with useful information for designing prevention and treatment programs, both at national and at state and municipal levels. The sample was composed of 27 geographic areas for the state capitals and the Federal District, and five for the remote areas of each region (North, Northeast, Midwest, Southeast, and South), totaling 32 domains. The primary sampling units were composed of: (a) municipality, for the remote regional areas, and (b) census tract, for the capitals. Individuals aged between 5 and 12 years, and those belonging to the age groups of 15–19, 35–44, and 65–74 years, were interviewed and examined in their homes.

The Community Periodontal Index (CPI) has been the most widely used index in Brazilian population surveys for the evaluation of periodontal condition. It was proposed by the WHO and is complemented by the periodontal attachment loss (PAL) exam for the adult and elderly population. Its function is to evaluate the occurrence of bleeding, calculus, and the presence of periodontal pockets (shallow and deep) using as reference the examination by sextant (groups of 6 teeth among the 32 of the dental arch) (9). The CPI level was used to identify the presence of bleeding and calculus in the 12-year-old group, and bleeding, calculus, and shallow (3–5 mm) and deep (6 mm or more) periodontal pockets in the groups representing adolescents (15–19 years), adults (35–44 years), and the elderly. Also, in adults and elderly, the PAL was measured by the PAL exam (9). To ensure a uniform and consistent standard for the collection of data interpretation criteria, professionals were trained to act as calibration instructors. These coordinators held workshops in each state, in order to calibrate the field teams, minimizing the variations between different examiners. The results of the calibration process, measured by percentage agreement and Kappa coefficient for different injuries and the age-indices contained in the calibration reports for each trained staff, were complemented by PIP (index of PAL) for adults and seniors. A minimum limit of agreement of 0.65 for weighted kappa is recommended for this study. The calibration procedure of each team took eight shifts of 4 h. Weighted Kappa values for agreement intra examiner from 0.69 to 0.87 for most examiners found. Good and excellent agreements were observed, with weighted Kappa ranging from 0.65 to 0.73 (CPI) for interrater concordance. Some critics referring to the use of this index occur due to limitations like no assessment of clinical attachment level, disregarding the history of tissue destruction as well as the low accuracy. In addition, CPI has limitations in assessing the situation of only a few teeth. Some authors argue that the CPI is an index developed for measurements of treatment need and that it is not useful for tracking changes in the prevalence of periodontitis. Despite its limitations in assessing the true extent and severity of the disease when used as the sole indicator index, it is advantageous due to its simplicity, ease, speed, and international uniformity in the application. For these reasons, it has been used in many descriptive epidemiological studies of periodontal disease in both developed and developing countries.

The 2010 survey revealed that the highest prevalence of 12-year-old children with periodontal disease was found in the North (58.4%) and the lowest in the Southeast (32.1%).

In the 15–19 years group, the North produces the worst periodontal conditions in this age group, in which only 30.8% of the adolescents in this region presented sextants in good health. The best conditions were observed in the Southeast, with 56.8% of the examined presenting healthy sextants.

In the North, we identified the highest prevalence of adults aged 35–44 years with periodontal disease (91.7%). And in the other regions, the percentages were very similar for the same phenomenon.

The worst periodontal conditions were identified in the elderly (65–74 years) in which only 1% of the sextants that could be evaluated were found healthy. Moreover, in this age group very few sextants could be evaluated, given the significant tooth loss in this population.

It was observed that the prevalence of gingival bleeding increases from the age of 12 years to adulthood, and decreases in case of the elderly. In Brazil, about a quarter of adolescents 12 years of age, one-third of adolescents aged 15–19 years, about half of adults aged 35–44 years, and less than one-fifth of the elderly have gingival bleeding. The presence of dental calculus increases with age, reaching the highest prevalence among adults, close to 64%, and declines in case of the elderly.

Major determinants of inefficiency in the less efficient regions (North and Northeast) are concentrated in the perspective of learning and growth (the number of health professionals and the number of graduates with a health-related undergraduate degree) and, in the regions with the best performance (South and Southeast) the major determinants of inefficiency are concentrated in the financial (spent on health care and the amount paid for hospitalization) (10).

Income, education, type of dental service most often used, lifestyle, risk behaviors, and demographic conditions are distal, intermediate, and proximal social determinants of health associated with functional dentition in adults (4). Various studies include socio-demographic variables in their analyses of the causes of periodontal disease, but few studies analyze the effect of contextual social determinant variables (1, 5, 11). The positions that social groups occupy are already associated with various health conditions. In a review of the use of social indicators in epidemiological studies on oral health, the findings were that the indicators used are heterogeneous, the ABA/ABIPEME indicator being the one most often used in Brazil, which analyzes economic potential and consumption habits (5).

A study analyzing data from the SB Brasil 2003 did not find any association between the Gini index of the cities studied and the severity of periodontal disease (12). Apparently, the more privileged socio-economic groups have a better understanding of morbidity and functional limitation; moreover, economic inequality modifies access to diagnostic methods; it is also associated with low education, and with the unequal distribution of services in a continental country like Brazil.

The decision to incorporate race in routinely collected health Brazilian statistics should take into consideration validity and reliability problems. The concept and classification of race are not uniform within datasets, among countries, or over time (13). Travassos and Williams showed significant demographic, socio-economic, and geographic variations across the categories of skin color/race (13).

It was found that the prevalence of severe periodontal disease, analyzed by the SB Brasil 2010 investigation, was higher in municipalities with higher income inequality. This inequality can affect access to health information and services networks, and it can also result in poor development of public policy. On the other hand, wider population coverage through the primary care strategy adopted in Brazil, incorporating oral health (Family Health Strategy/Oral Health Teams), was associated with better indicators of periodontal health (1).

The cross-sectional nature of the studies pointed out here limit, in part, the capacity to draw conclusions from the data. However, they are capable of showing the improvements in the epidemiological status of the population, although they continue to indicate differences between the various regions of the country.

## Conclusion

We conclude that oral health surveys, particularly on periodontal disease, population based and conducted in developing countries with continental dimensions, are challenges for health surveillance. Ecological and multilevel studies have, in this setting, presented an important step in the advancement of oral health epidemiology, allowing a more contextualized formulation and assessment of health policies.

## Conflict of Interest Statement

The authors declare that the research was conducted in the absence of any commercial or financial relationships that could be construed as a potential conflict of interest.
